# Dysregulation of the peripheral glutamatergic system: A key player in migraine pathogenesis?

**DOI:** 10.1177/03331024211017882

**Published:** 2021-06-20

**Authors:** Tarique Benbow, Brian E Cairns

**Affiliations:** Faculty of Pharmaceutical Sciences, University of British Columbia, Vancouver, Canada

**Keywords:** Glutamate, migraine, peripheral sensitization, neurogenic inflammation

## Abstract

**Background:**

Although the role of glutamate in migraine pathogenesis remains uncertain, there has been significant interest in the development of drug candidates that target glutamate receptors. Activation of trigeminovascular afferent fibers is now recognized as a crucial step to the onset of a migraine episode. New evidence suggests a dysfunction in peripheral glutamate regulation may play a role in this process.

**Objective:**

To provide a narrative review of the role of peripheral glutamate dysfunction in migraine.

**Method:**

A review of recent literature from neurobiological, pharmacological and genomic studies was conducted to support peripheral glutamate dysfunction as a potential element in migraine pathogenesis.

**Results:**

Studies in rats suggest that elevated blood glutamate mechanically sensitizes trigeminal afferent fibers and stimulates the release of calcitonin-gene related peptide and other neuropeptides to promote and maintain neurogenic inflammation. These effects may be driven by upregulation of glutamate receptors, and modifications to reuptake and metabolic pathways of glutamate. Furthermore, genome wide association studies have found polymorphisms in glutamate receptor and transporter genes that are associated with migraine.

**Conclusion:**

The role of peripheral glutamate signalling in the onset and maintenance of migraine is not completely elucidated and future studies are still needed to confirm its role in migraine pathogenesis.

## Introduction

Activation of the trigeminovascular system is a major pathological step in the initiation of a migraine attack (1). However, exactly how this system is activated remains uncertain. Glutamate, an endogenous excitatory amino acid neurotransmitter present in the central and peripheral nervous systems has been implicated as a contributing factor in this process (2–4). Preclinical and clinical studies provide evidence of the role glutamate plays in migraine pathogenesis and have consequently led to the development of drugs targeting glutamate receptors. These drug candidates were specifically designed to target central glutamate receptors but failed to provide superior efficacy when compared to current therapies or produced unacceptable centrally mediated adverse effects (2,3,5). Furthermore, given the recent recognition of the importance of calcitonin-gene related peptide (CGRP) in migraine headache pathogenesis, attention has been focused away from the role of the glutamatergic system in migraine. Nevertheless, new insights into migraine pathogenesis, glutamate signalling, receptor expression and their influence on the peripheral structures involved in migraine pathogenesis may re-ignite this interest.

Scientific evidence supports the notion of an imbalance of glutamate regulation within the peripheral nervous system in migraine attacks. For example, serum levels of glutamate have been found to be elevated in migraine patients particularly after a migraine attack (6,7). Meanwhile, studies in rats have found that elevated levels of glutamate lowered the mechanical force required to excite trigeminovascular neurons and increased dural blood flow (8). These findings may indicate the potential for dysregulation of glutamatergic system in the periphery to contribute to migraine pathogenesis. This narrative review discusses recent findings from pharmacological, neurobiological and genomic studies on the role of glutamate signalling in craniofacial pain mechanisms.

## Current understanding of pain generation in migraine

It is thought that peripheral sensitization of the trigeminovascular system is key to the onset of a migraine attack (9,10). Peripheral sensitization results in increased nociceptor excitability. It is driven by the release of vasoactive substances including neuropeptides such as CGRP, substance P and, possibly, glutamate, from afferent fiber endings that innervate the dura and its blood vessels (11,12). In addition to vasodilation, release of these substances leads to plasma protein extravasation and release of other inflammatory mediators (kinins, amines, prostaglandins, growth factors, chemokines, cytokines, protons, adenosine triphosphate and glutamate) from surrounding tissues, which results in a process known as sterile neurogenic inflammation (13,14). These inflammatory mediators can further reduce afferent activation threshold and increase afferent responsiveness to noxious stimuli (sensitization) (15,16).

The intensity and duration of a migraine attack is thought, in part, to be due to the development of central sensitization. Central sensitization is described as an abnormal amplification in central nociceptive processing, and results in the spread of the painful area beyond the original site of injury (18). The initial changes that lead to central sensitization in response to noxious stimulation of craniofacial tissues, including the dura, involve an increased response to synaptically released glutamate as a result of phosphorylation of N-methyl-D-aspartate (NMDA) receptor subunits (19–22). Altered transmission within the trigeminal neurovascular system can also result in a decrease in descending inhibition and/or enhanced descending facilitation of nociception (23,24). This has numerous implications within the context of the development of chronic migraine, where the lowered sensory threshold from recurrent migraine attacks is argued to be mediated by activity-independent sensitization of central trigeminothalamic pathways (12). Central sensitization is driven by neuroplasticity, which occurs due to recurrent intense sensory inputs from the periphery (25,26). These neuroplastic alterations in trigeminovascular neurons include a reduction in threshold, exaggerated response to noxious stimulus and increase in spontaneous firing, which ultimately leads to increase pain intensity and duration. In this regard, glutamate is thought to play an integral role in contributing to these neuroplastic changes. One possibly is through downstream upregulation of NMDA and α-amino-3-hydroxy-5-methyl-4-isoxazolepropionic acid (AMPA) receptors on primary afferent endings, thereby modifying activation threshold and the development of central sensitization (27).

In addition to its action on central sensitization, other central glutamatergic mechanisms have been proposed in migraine pathogenesis. Specifically in migraine with aura, the aura symptom is thought to be explained by cortical spreading depression (CSD) (28). Cortical spreading depression is a wave of depolarization of neuronal and glial cells followed by a sustained suppression of spontaneous neuronal activity (28). Central glutamate mechanisms are thought to play a role in this phenomenon (29,30). Local release of glutamate by neurons is thought to initiate CSD and the subsequent activation of post-synaptic central glutamate receptors is argued to explain its propagation (29–31). Furthermore, inhibition of CSD by memantine, an NMDA receptor antagonist, also suggests a key role for activation of neuronal glutamate receptors in the initiation of CSD (32). In preclinical studies, tonabersat, a gap-junction inhibitor, has also been shown to reduce and inhibit CSD (33). Despite the ability of tonabersat in inhibiting aura, tonabersat showed no advantage in migraine headache relief (33,34). This suggests that migraine aura may be dissociated from the development of migraine headaches. Therefore, while central glutamatergic mechanisms are implicated in both aura and central sensitization, the prophylactic use of centrally acting glutamate receptor antagonists for migraine is limited by central nervous system (CNS) adverse effects. Since peripheral glutamate receptor mechanisms are proposed to be involved in migraine headache pathogenesis and inhibition of these receptors would be less likely to lead to CNS adverse effects, drugs that selectively inhibit these receptors would be of interest (12,35).

## Peripheral glutamatergic pharmacology

The functional role of glutamate in sensory transmission provides the first hint of its importance in migraine pathogenesis. Two main types of glutamate receptors exist: ionotropic and metabotropic glutamate receptors. It should be noted that only details relevant to migraine are covered in this review as the structure and function of glutamate receptors have been more comprehensively appraised in previous literature (36,37).

## Inotropic glutamate receptors

The ionotropic glutamate receptors are subdivided into N-methyl-D-aspartate (NMDA), α-amino-3-hydroxy-5-methyl-4-isoxazole propionic acid (AMPA) and kainate receptors (38). Ionotropic glutamate receptors are involved in fast synaptic signalling and thus are vital to the relay of nociceptive input from the periphery to the brain. Interestingly, all three ionotropic glutamate receptors have been characterized and found to be constitutively expressed in trigeminal ganglion neurons (8). However, while the expression of ionotropic glutamate receptors on trigeminal ganglion neurons that innervate the dura-mater specifically has not been characterized, NMDA receptors are expressed by afferent fibers that innervate the dura blood vessels (8). The role these peripheral receptors play in craniofacial pain conditions that include migraine needs to be better understood.

**Figure 1.**
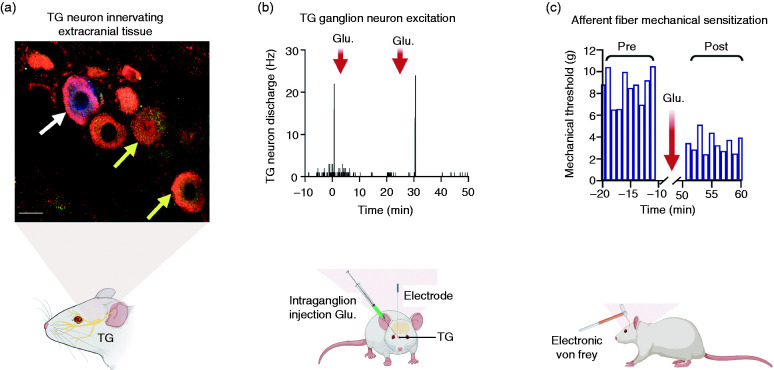
(a) The image is of trigeminal ganglion neurons that express the NR2B subunit of the NMDA receptor (red). The white arrow indicates a neuron that projected to the ipsilateral temporalis muscle (blue). The yellow arrows indicate trigeminal ganglion neurons that expressed both the NR2B subunit and CGRP (green) (unpublished data). (b) The peristimulus histogram shows the response of a trigeminal ganglion neuron that innervated the temporalis muscle to intraganglionic injection of glutamate (500 mM, 0.3 µl) at 0 min and 30 min. Responses to glutamate were relatively reproducible. (c) The bar chart shows the afferent mechanical activation threshold before and after repeated intraganglionic administration glutamate shown in (b). The mechanical activation threshold from the temporalis muscle was decreased by 60%. This afferent fiber was recorded in a female rat and had an estimated conduction velocity of 5.2 m/s (data from (75)) (created with personal license of BioRender).

NMDA receptors are tetrameric ion channels composed of two GluN1 subunits and two GluN2 subunits or two GluN3 subunits, although tetramers containing GluN3 subunits are not functional (39,40). NMDA receptors are permeable to calcium (Ca^2+^) and monovalent cations (K^+^, Na^+^) (41). There are four types of GluN2 subunits: GluN2A, GluN2B, GluN2C and GluN2D (42). Two (2) glutamate molecules must bind to sites on the GluN2 subunits for receptor activation (43). NMDA receptors also possess regulatory sites that require the binding of the co-agonists, glycine and/or d-serine to the GluN1 subunit (43). Still, at resting potential, the receptor channel is blocked by an Mg^2+^ ion and so the membrane potential must be sufficiently depolarized to repel the Mg^2+^ from the channel to allow channel opening (40,44). This makes NMDA receptors voltage dependent (43) and highlights the complexities of their gating mechanism and role in synaptic tuning. Intriguingly, NMDA receptors have been shown to be expressed in trigeminal ganglion neurons, including those that transmit noxious stimuli (45) (Figure 1(a)). Of the NMDA receptors expressed in these neurons, the GluN1-GluN2B subunits are the most abundantly expressed (42). Intraganglionic injection of glutamate can excite trigeminal ganglion (TG) neurons through activation of NMDA receptors (Figure 1(b)). Preclinical studies suggest that the activation threshold of trigeminovascular neurons to mechanical stimulation of the dura is decreased after intravenous administration of monosodium glutamate, which is restricted to the vascular compartment (8). This effect was blocked by 5-aminophosphonovaleric acid (APV; a NMDA receptor antagonist), which also does not effectively cross the blood-brain barrier. These findings suggest that peripheral NMDA receptor activation may play a role in regulating the sensitivity of the trigeminovascular pathway, but it is unclear if their activation contributes directly to the initiation of a migraine attack.

The majority of fast excitatory neurotransmission that occurs in the central nervous system is mediated by AMPA receptors (46). Like NMDA receptors, AMPA receptors are tetramers consisting of one or more GluA1-4 protein subunits (47,48). These receptors respond to glutamate, which results in opening of a cation conducting channel and depolarization of the post-synaptic membranes (49). Associated with each AMPA receptor are four types of auxiliary subunits (TARP, GSGL1, Cornichon, CKamp/Shisa), which regulate the trafficking, gating, pharmacology, and ion permeation of these receptors (47,50,51). AMPA receptors are expressed abundantly in the peripheral nervous system, including the trigeminal afferent endings, axons and ganglion somas (52). However, not very much is known about the role of peripheral AMPA receptors play in pain transmission and their contribution to the onset of a migraine attack.

Kainate receptors are a tetrameric assembly of subunits and are structurally similar to their NMDA and AMPA counterparts (53). There are five receptor subunits that may assemble to form the tetramer unit: GluR5, GluR6, GluR7, KA1 and KA2 (54,55). Kainate receptor subunit RNAs (GluR5 and KA2 containing receptors) are expressed in the TG of rats but to a lower extent compared to NMDA and AMPA receptors (52,56). Nevertheless, their presence in the TG may have functional implications in migraine pathophysiology. Interestingly, the expression of kainate receptors within trigeminal ganglion neurons has been shown to increase after an injection of nitroglycerin (10 mg/kg), which is used in preclinical models of migraine (57). Another study also found that activation of GluR5 kainate receptors inhibited neurogenic dural vasodilation produced by electrical stimulation of the dura mater (58). Since CGRP and glutamate release is mediated by calcium channels, a possible explanation of how kainate receptors may mediate these effects is through pre-synaptic inhibition of calcium and sodium channels at the primary afferent endings (56,59). However, given the low expression of kainate receptors in TG neurons compared to their NMDA and AMPA counterparts, it is unlikely that their activation alone results in sufficient inhibition to affect the development of a migraine episode. Additional research is needed to elucidate the exact mechanisms and contributory role kainate receptors play in these processes.

## Metabotropic glutamate receptors

Metabotropic glutamate receptors (mGluR) are G-protein-coupled receptors that are responsible for slow neuromodulatory responses to glutamate (60). They are divided into three groups based on their amino acid sequence homology (61). Group I (mGluR1 and mGluR5), Group II (mGluR2 and mGluR3) and Group III (mGluR4,6-8). Each group displays differences in downstream signalling of intracellular second messengers (61). Group I metabotropic glutamate receptors (mGluR1 and mGluR5) are coupled to Gq which, upon activation, leads to an increase in inositol triphosphate (IP3) and diacyl glycerol (DAG) and ultimately cytosolic Ca^2+^ levels. The functional outcome of activation of these receptors on the cell is excitatory; mechanisms include promotion of NMDA receptor migration to the cell membrane, increasing NMDA receptor conductance and enhanced vesicular release of glutamate from the presynaptic nerve endings (62,63). TG neurons along with their associated satellite glial cells have been shown to express mGluR1_a_ subtypes specifically (64). Other studies have found a functional role for group 1 mGluRs on sensory processing within the periphery and thus a link to painful conditions (65). One study revealed that stimulation of mGluR1 in the muscles of rodents is nociceptive (66). Likewise, local inflammation using injection of complete Freud’s adjuvant (CFA) into the masseter muscle of rodents yielded an increased expression of mGluR5 receptors in the TG (67).

Group II and group III mGluRs are coupled with Gi protein, which inhibits the activation of adenylyl cyclase and leads to a subsequent reduction in intracellular cyclic adenosine monophosphate (cAMP) concentrations (68). The functional effect of group II and III mGluR activation on the neuron is largely inhibitory; reducing conductance of L-type Ca^2+^ channels and postsynaptic potentials by inhibiting vesicular release of glutamate (69). Several studies have examined the functional effects of the activation of group II and III mGluRs with respect to sensory processing in the periphery. Group II mGluRs have been shown to be co-expressed with transient receptor potential channel vanilloid 1 (TrpV1) receptors on peripheral nociceptors and upon activation inhibit painful sensory transmission mediated by TrpV1 activation (70). Similarly, it has been revealed that stimulation of group II and III mGluRs hyperpolarizes the endings of trigeminal afferent fibers to inhibit their spontaneous discharge (71). Activation of group II mGluRs have also been shown to reduce interlukin-1β (IL-β)-induced mechanical allodynia in the orofacial region in conscious rodents (72). The current evidence suggests that peripheral metabotropic glutamate receptors have the potential to mediate several components of sensory processing within the trigeminovascular neurons, which could include neurogenic inflammation, and therefore warrant further research into their role in migraine pathogenesis.

## Glutamate transporters

Glutamate transporters are synaptic transmembrane proteins that play a fundamental role in the regulation of extracellular glutamate levels in the central and peripheral nervous system. They cluster in high densities in the plasma membrane and contribute to inactivation of glutamate signaling. There are five excitatory amino acid transporters (EAAT 1-5) that are responsible for the uptake of glutamate from the extracellular space into cells: Glutamate/aspartate transporter (GLAST = EAAT1), glutamate transporter 1 (GLT1 = EAAT2), excitatory amino acid carrier (EAAC1 = EAAT3), excitatory amino acid transporter 4 (EAAT4), and excitatory amino acid transporter 5 (EAAT5) (73). The GLT-1 transporter is responsible for approximately 90% of glutamate reuptake from the synaptic cleft and maintains peripheral glutamate levels between 30–80 µM (73). In the central nervous system, GLT-1 and GLAST are expressed constitutively on astrocytes and microglia and are upregulated following spinal nerve ligation in the rat (74). Satellite glial cells (SGCs) that form functional units with trigeminal ganglion neurons also express glutamate transporters and help to regulate extracellular glutamate levels in the ganglion (75). The functional consequence of glutamate transporter blockade in the TG has also been studied to some extent. One study revealed that inhibition of glutamate uptake by SGCs in the trigeminal ganglion increased neuronal discharge in response to glutamate (75). A dysfunction of glutamate transporters within the trigeminal ganglion and in other tissues could contribute to the peripheral sensitization that is thought to underlie migraine pathogenesis.

Vesicular transporters VGluT1 and VGluT2 permit the uptake of glutamate into synaptic vesicles and have also been shown to be co-expressed in the cell bodies and axons terminals of most trigeminal ganglion neurons including nociceptors that transmit noxious stimuli signals to the central nervous system (76). VGluT3 has been characterized in a smaller subset of ganglion neurons whose axons are unmyelinated (77). The expression of glutamate transporters by trigeminal afferent fibers suggests that glutamate is contained within vesicles at afferent terminal endings and released under conditions that lead to neurogenic inflammation.

## The role of glutamate in trigeminovascular system: Activation, sensitization and neurogenic inflammation

**Figure 2.**
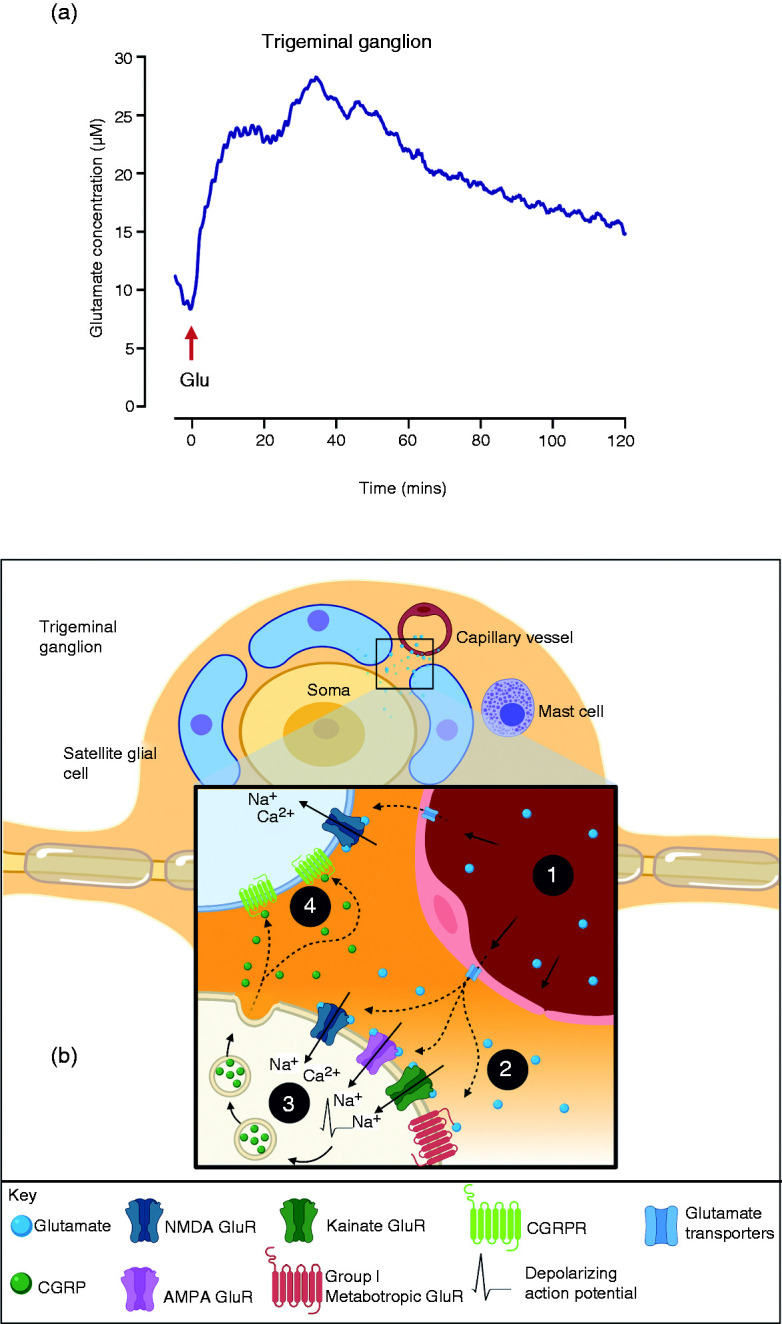
(a) The line graph illustrates how glutamate concentration is altered in the trigeminal ganglion after exogenous administration of MSG (unpublished data). Glutamate concentration was measured in the trigeminal ganglion of an anesthetized male rat with a glutamate selective biosensor (Pinnacle Technology Inc., USA) before and after administration of 50 mg/kg monosodium glutamate (MSG). During the 5-min baseline the average glutamate concentration was 9.5 µM. MSG was injected into the carotid artery at time 0. There was a prolonged increase in trigeminal ganglion glutamate concentration after injection that approached a peak of just under 30 µM, which suggests that the concentration of glutamate in the trigeminal ganglion can be increased by exogenous administration of MSG. (b) The diagram illustrates how exogenous and endogenous glutamate could affect trigeminal ganglion neuron function. 1. Plasma glutamate enters the trigeminal ganglion from capillaries or is released by satellite glial cells and diffuses to the soma of trigeminal neurons. 2. Glutamate binds to and activates glutamate receptors (NMDA, AMPA, Kainate and mGluR) expressed on trigeminal ganglion cell membrane. 3. The influx of cations depolarizes the cell membrane and triggers the release of neuropeptides (CGRP, Substance P, etc.). 4. CGRP activates satellite glial cells which release additional glutamate to maintain glutamate signalling and sensitization in a vicious cycle (created with personal license of BioRender).

The trigeminal ganglion soma are surrounded by satellite glial cells, collagen fibers and capillary blood vessels and are not protected by the blood-brain barrier (78–80). This therefore suggests that TG function is susceptible to changes in blood chemistry (81,82). Indeed, exogenous administration of monosodium glutamate (MSG) (50 mg/kg) can be shown to substantially elevate glutamate levels in the TG (Figure 2(a)). Intra-ganglionic injection of glutamate evokes ganglion neuron discharge and can also mechanically sensitize the peripheral endings to trigeminal afferent fibers (Figure 1(b),(c)) (75). SGCs express excitatory amino acid transporters (EAATs), and it has been demonstrated that ganglion neuron discharge evoked by intra-ganglionic injection of glutamate is increased by (3S)-3-[[3-[[4-(trifluoromethyl)benzoyl]amino]phenyl]methoxy]-L-aspartic acid (TFB-TBOA), an EAAT1,2 inhibitor (75). Further, SGCs contain glutamate (75). Depolarization of SGCs results in the vesicular mediated release of glutamate, which could then diffuse to affect trigeminal ganglion neurons (Figure 2(b)) (75,83). These findings suggest that increased glutamate concentrations in the trigeminal ganglion could, therefore, lead to sensitization of facial skin and cranial muscle afferent fibers, which could be perceived as skin and muscle sensitivity. Muscle and skin sensitivity are common features associated with migraine headaches, although these have generally been thought to reflect central nervous system mechanisms.

Preclinical studies have provided evidence that glutamate can activate and sensitize trigeminal afferent fibers. In rats, intravenous administration of 50 mg/kg of MSG increased masseter muscle interstitial glutamate concentrations from a baseline of around 20 µM to about 60 µM and induced mechanical sensitization of afferent fibers that innervate this muscle (8,68). Still, other studies revealed that activation of ionotropic and group I metabotropic glutamate receptors on sensory neurons produce nociception (85,86). In healthy humans, baseline plasma glutamate is around 35 µM (87–89). Thirty minutes after oral administration of 150 mg/kg of MSG, plasma glutamate levels increase to around 100 µM, and in many subjects are associated with reports headache and craniofacial muscle sensitivity. In a small group of migraine patients without aura, plasma glutamate levels were reported to be, on average, 60 µM (90). These findings suggest that a 3–4 times increase of plasma glutamate levels can result in peripheral sensitization, and in healthy individuals may lead to reports of headache.

**Figure 3.**
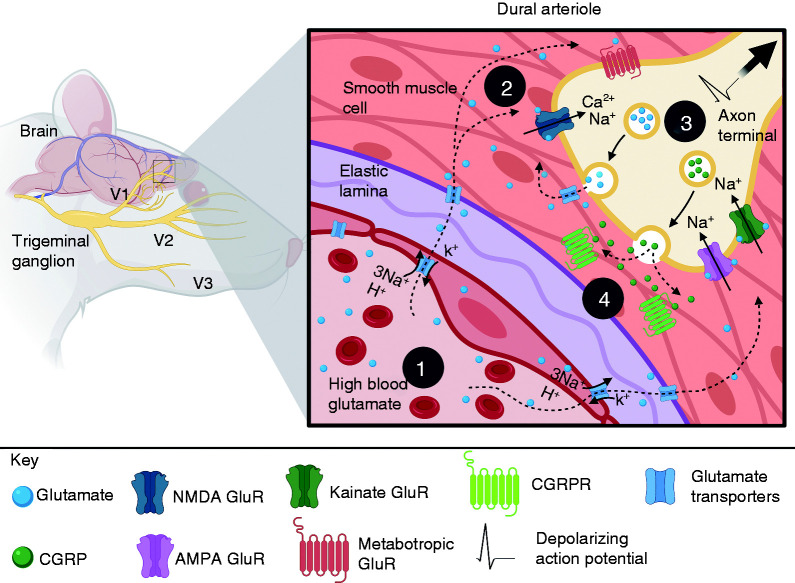
A proposed mechanism through which peripheral glutamate may activate dural afferent fibers to induce neurogenic inflammation. 1. Glutamate is taken up from the plasma into dural vascular epithelium by GLT1 (EAAT 2) (8). 2. Glutamate binds to and activates peripheral glutamate receptors (NMDA, AMPA, Kainate and group I mGluR) located on trigeminal afferent fibers that innervate the vascular epithelium. 3. Influx of Ca^2+^ and Na^2+^ depolarizes the axonal membrane to induce afferent sensitization and trigger the vesicular release of neuropeptides (CGRP and Substance P). 4. CGRP and substance P bind to arteriole smooth muscle cells, producing vasodilation. Substance P also increases postcapillary vascular permeability (not shown), which allows plasma glutamate to access afferent endings in addition to other blood algogens (5HT and ATP), thereby driving dural afferent sensitization (created with personal license of BioRender).

Plasma glutamate may play a role in the initiation and maintenance of sterile neurogenic inflammation in the dura. Immunohistochemistry studies illustrate that rat TG neurons, which innervate the masticatory muscles, express NMDA receptors and contain CGRP and substance P (Figure 1(a)) (91). Afferent fibers of the dural meningeal arteries, as well as many trigeminal ganglion neurons, express both CGRP and substance P (92,93). The nerve fibers that innervate the rat dural vasculature also express NMDA receptors, and intravenous administration of MSG increases dural blood flow and lowers the threshold for trigeminovascular neuron response to mechanical stimulation of the dura (8). Functional studies have demonstrated that TG afferent fibers release CGRP and substance P after activation of glutamate receptors (15). For example, injection of MSG into the masseter muscle produces afferent mechanical sensitization through activation of NMDA receptors and vasodilation through CGRP and substance P (94). We propose that elevated plasma levels of glutamate could act on NMDA receptors expressed by dural vascular afferent fibers to release CGRP and substance P and produce neurogenic inflammation. This suggests that elevations in plasma glutamate may not only promote neurogenic inflammation but may also maintain this process. This could lead to further sensitization of these fibers in a vicious cycle (Figure 3). Future studies will be needed to confirm this hypothesis.

## Structural modifications and changes in the expression of peripheral glutamate receptors and transporters

Increased expression of NMDA and AMPA receptors in trigeminovascular neurons might also lower the afferent activation threshold and contribute to migraine initiation. Studies show that inhibiting peripheral AMPA and NMDA receptors decreases pain behaviour and nociception in rats with masseter muscle myositis (95,96). Injection of nerve growth factor (NGF) has been shown to induce mechanical sensitization and increase the expression of NMDA receptors within trigeminal afferent fibers that innervate the masseter muscle (97). This upregulation of NMDA receptors by NGF injections followed a sexually dimorphic pattern, where greater sensitization was observed in slowly conducting A-delta fibers in female rats compared with male rats (97). The sex differences seen in the effect of NMDA receptor activation and expression in rats is parallel to the sex differences observed in migraine occurrence in humans.

Few studies have specifically examined the functional consequences of peripheral glutamate receptor morphology changes and how these changes influence sensory transmission though the TG. Structural changes that increase glutamate binding affinity to its inotropic receptors could potentially lower the activation threshold and heighten sensory signalling in the TG. Post-translational phosphorylation of AMPA receptors yields lower affinity for glutamate and is mediated by NMDA receptor mechanisms (98), while phosphorylation of the NMDA receptor enhances receptor activity and function as discussed above (99). This is demonstrated in “pain states,” where the NMDA receptor is phosphorylated as a mechanism to enhance receptor function (100). It is plausible that increased phosphorylation of peripheral NMDA receptors might increase neuronal excitation in the trigeminovascular afferent fibers and thus lower activation thresholds. Studies have yet to be conducted to confirm if this phenomenon occurs and if there are functional implications to the development of a migraine.

Alterations in the expression and function of peripheral glutamate transporters could also have detrimental effects on trigeminal sensory relays. However, it is unknown what structural change or variation in expression levels of glutamate transporters could result in functional deficits within peripheral structures relevant to migraine. Nevertheless, studies from other neurological diseases provide clues into how this might be possible. Glycosylation of EAAT1 and EAAT2 is crucial to their ability to bind to glutamate. One study demonstrated that less glycosylation of EAAT1 and EAAT2 leads to increased neuronal excitation in schizophrenic patients (101). Although not confirmed in migraine-related structures, it is possible that low glycosylation of EAAT1 and EAAT2 transporters could contribute to lower glutamate affinity and clearance and thus prolong trigeminal neuron activation. This is an area yet to be explored in migraine research.

To this end, glutamate oxaloacetate transaminase (GOT), an endogenous enzyme that catalyses the reversible conversion of L-aspartate to glutamate and vice versa, has been demonstrated to be reduced in migraine patients with elevated levels of blood glutamate (102). A deficit in this enzyme might lead to dysfunction in amino-acid metabolism, including increased blood glutamate concentrations, which could lead to sensitization of trigeminovascular afferent fibers.

## Genome-wide association studies implicate peripheral glutamate dysfunction in migraine

Genome-wide association studies (GWAS) have identified a potential dysregulation of glutamate reuptake mechanisms in migraine. Polymorphisms in the glutamate transporter EAAT2 have been identified in some people living with chronic migraine, and are associated with a tendency towards increased usage of analgesics in these patients (103). The EAAT2 transporter expression is regulated by the metadherin (MTDH) gene (a gene involved in glutamate homeostasis) (104). One study found that a polymorphism in the promoter of the MTDH gene was associated with higher sustained plasma glutamate concentrations together with higher frequency of neurological deterioration in patients with acute hemispheric stroke (103). Furthermore, a meta-analysis of GWAS studies in migraine patients revealed a modest gene-based association between the MTDH gene and migraine (105). It is therefore possible that polymorphisms of the MTDH gene could decrease the expression of EAAT2 transporters and alter glutamate metabolism in the periphery. The full potential of an association between EAAT2 glutamate transporter polymorphisms and migraine as well as the functional implications on sensory transmission is yet to be confirmed.

Other GWAS studies have identified SNPs in migraine patients that are associated with glutamate receptor expression and function. One population-based GWAS study in women indicated a significant association between the rs11172113 (2q13.3, LDL Related Receptor-1(LRP1)) SNP and migraine (106). They found that the LRP1 are co-expressed with NMDA receptors and postulated that polymorphisms in these receptors may influence glutamate receptor functionality (106). Similarly, one study examined the association of GRIA1-GRIA4 genes (which encode for the four subunits of the AMPA receptors; GluR1–4) in migraine with and or without aura (107). They discovered that the rs548294 variant of the GRIA1 gene was associated primarily with the migraine without aura phenotype (107). These GWAS studies suggests a possible link between abnormal glutamate signalling and specific phenotypes or symptoms of migraine.

## Clinical implication of peripheral glutamate signalling in migraine

NMDA receptor antagonist drugs such as ketamine and memantine have been investigated for migraine treatment and prophylaxis, respectively (108–110). Several studies have also investigated the use of NMDA (ketamine) or AMPA (LY293558; BGG492) antagonists as abortive therapies in migraine with aura and familial hemiplegic migraine (5,108,111,112). Although most of these studies concluded that these drugs possessed limited or at best moderate efficacy in reduction of severity of headaches and analgesic consumption, the presence of side effects owing to their effects on central glutamate receptors limited future exploration of their use in migraine (2). In this regard, peripherally restricted glutamate receptor antagonists may offer an alternative with a lower potential for adverse effects. It has been proposed that targeting the glutamatergic system does not play a decisive role once migraine has begun (3). This suggests that targeting peripheral glutamate receptors might be most beneficial in migraine prophylaxis therapy. The effectiveness of memantine as a migraine prophylactic hints at the potential usefulness of novel glutamate receptor antagonists for migraine prophylaxis (110,113).

Elucidation of the role of peripheral glutamate signalling in migraine may also benefit non-pharmacologic approaches to managing migraine. MSG is the third most common dietary trigger for headaches. In healthy people, ingestion of 150 mg/kg MSG has been shown to increase reports of headache, nausea, and vomiting, symptoms also associated with acute migraine attacks (84). In fibromyalgia patients, reduced dietary MSG intake has been associated with reduction in musculoskeletal pain (114). This suggests that, at least for some people living with migraine, initiation of a diet low in MSG may be effective in preventing attacks. In children with primary headaches, exclusion of dietary headache triggers, which included MSG, was reported to decrease headaches (115). While future studies are needed to investigate the benefit of a low MSG diet in migraine, diets low in MSG are available and have few if any negative consequences. They should be considered as part of a migraine prophylactic regimen in patients who report perceived MSG sensitivity.

## Concluding remarks and future perspective

Preclinical evidence suggests that the peripheral glutamatergic system might be a target for migraine prophylactic therapy (2,8). This opens up the possibility of conducting clinical trials to investigate glutamate receptor antagonists that are restricted to the periphery as prophylactic migraine treatments. Furthermore, there is an opportunity to develop a novel class of glutamate receptor modulators that could target specific glutamate receptor subtypes and/or glutamate transporters based on expression levels in the trigeminovascular primary afferent fibers. One possible target, based on preclinical studies, is the NR2B containing NMDA receptors, as these have been shown to be highly expressed in trigeminal ganglion neurons and their surround cerebral blood vessels (8). Additionally, modulation of glutamate transporters is a potential therapeutic approach yet to be examined. Further investigation into peripheral glutamatergic dysfunction is needed to provide support for glutamate as a key player in migraine.

## Key findings

- Elevated plasma glutamate levels may trigger migraine and perpetuate neurogenic inflammation.
- Peripheral glutamate receptors and regulation of glutamate levels may be potential new targets for migraine prophylactic agents.
- Further evidence is required to reveal and confirm the function of peripheral glutamate dysfunction in migraine.
